# Low Temperature Synthesis of Belite Cement Based on Silica Fume and Lime

**DOI:** 10.1155/2014/873215

**Published:** 2014-10-29

**Authors:** M. A. Tantawy, M. R. Shatat, A. M. El-Roudi, M. A. Taher, M. Abd-El-Hamed

**Affiliations:** ^1^Chemistry Department, Faculty of Science, Minia University, Minia 61111, Egypt; ^2^Chemistry Department, Faculty of Science, Al-Azhar University, Assiut 71524, Egypt

## Abstract

This paper describes the low temperature synthesis of belite (*β*-C_2_S) from silica fume. Mixtures of lime, BaCl_2_, and silica fume with the ratio of (Ca + Ba)/Si = 2 were hydrothermally treated in stainless steel capsule at 110–150°C for 2–5 hours, calcined at 600–700°C for 3 hours, and analyzed by FTIR, XRD, TGA/DTA, and SEM techniques. Dicalcium silicate hydrate (hillebrandite) was prepared by hydrothermal treatment of lime/silica fume mixtures with (Ca + Ba)/Si = 2 at 110°C for 5 hours. Hillebrandite partially dehydrates in two steps at 422 and 508°C and transforms to *γ*-C_2_S at 734°C which in turn transforms to *α*′-C_2_S at 955°C which in turn transforms to *β*-C_2_S when cooled. In presence of Ba^2+^ ions, *β*-C_2_S could be stabilized with minor transformation to *γ*-C_2_S. Mixture of silica fume, lime, and BaCl_2_ with the ratio of (Ca + Ba)/Si = 2 was successfully utilized for synthesis of *β*-C_2_S by hydrothermal treatment at 110°C for 5 hours followed by calcination of the product at 700°C for 3 hours.

## 1. Introduction

Portland cement is a hydraulic binder forming a paste with water, which sets and hardens due to hydration reactions [1]. The main constituent of Portland cements is clinker, which itself is composed of 40–80 wt.% tricalcium silicate 3CaO·SiO_2_ (alite, C_3_S), 10–50 wt.% dicalcium silicate 2CaO·SiO_2_ (belite, C_2_S), 0–15 wt.% tricalcium aluminate 3CaO·Al_2_O_2_ (aluminate, C_3_A), and 0–20 wt.% tetracalcium aluminoferrite 4CaO·Al_2_O_3_·SiO_2_ (ferrite, C_4_AF) [2]. Alite and belite determine most of the adhesive properties, strength, and durability of Portland cement. Both alite and belite show the same physical and mechanical properties after complete hydration but the former hydrates much faster [3]. Cement industry is responsible for approximately 5% of total CO_2_ emissions [4]. About 900–1000 kg CO_2_ per tonne of clinker is released due to calcination of CaCO_3_ and fuel combustion [2, 5]. The demand for thermal energy equals ~3000–4000 MJ/tonne of clinker for dry process [2]. Additionally, ~324–540 MJ/tonne of cement of electrical energy is required for grinding of raw materials and cement [6]. The production temperature of belite is lower than that of alite. In contrast, belite has low reactivity than alite, rendering it much less useful. The high reactivity of alite is related to the reactive sites around its more ionic oxygen atoms that are not contained in belite [7]. Attempt to improve the reactivity of belite phase in order to reduce energy requirements for cement production is one of the most important challenges.

**Figure 1:**
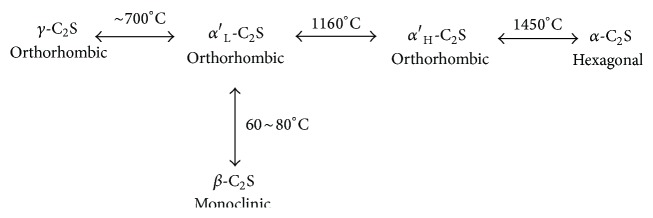
Polymorphs of belite.

Belite has five polymorphs (*β*, *γ*, *α*, *α*_H_′, and *α*_L_′) undergoing reversible temperature dependent transformations as illustrated in Figure 1 [8]. The *α*_H_′, *α*_L_′, and *β* polymorphs are derived from the *α* form by a decrease of the symmetry due to the disorder of SiO_4_^−4^ groups and slight changes in the position of Ca atoms [9]. The *α*′-polymorphs are the most hydraulic forms of belite. *β*-belite is also a hydraulic but less hydraulic than the *α*′-polymorphs. *γ*-belite is a nonhydraulic polymorph and does not account for the setting and hardening of cement. *β*-belite is the most common polymorph in industrial Portland cement clinker [10]. *γ*-C_2_S crystals are less dense (more voluminous) than *β*-C_2_S crystals, which causes cracking of other *β*-C_2_S crystals, forming voluminous powder and dust. Dusting phenomenon can be prevented if *β*-C_2_S is stabilized by fast cooling and/or by inclusion of stabilizing ions. Fe^3+^, Al^3+^, Mg^2+^, Zn^2+^, Cr^3+^, Pb^2+^, B^3+^, Na^+^, and K^+^ can replace calcium and/or silicon atoms in the structure and reduce the Ca/Si ratio of *β*-C_2_S to less than 2 [11, 12]. Reactivity and hydraulic properties of stabilized *β*-C_2_S depend on the type and amount of the stabilizing ions [13]. On the other hand, fast cooling may produce fine *β*-C_2_S crystals affording *β*-*γ*-C_2_S transformation, even without the need for stabilizer [14]. Based on density functional theory calculations, stabilizing ions modify charge density localization of the electronic structure of C_2_S and enhance the reactivity and in turn would broaden the applicability of C_2_S [7].

Synthesis of low-energy belite cement as an alternative to the conventional Portland cement was carried out in two steps. The first step is preparation of dicalcium silicate hydrate (hillebrandite, Ca_2_(SiO_3_)(OH)_2_) by hydrothermal treatment of silica rich waste material/lime mixtures. Hillebrandite was synthesized by hydrothermal treatment of quartz and lime with Ca/Si = 2.0 at 200°C for 10 hours or at 250°C for 5 hours [15]. The main silicate anion structure of hillebrandite studied by ^29^Si MAS NMR analysis is a mixture of a dimer and a single-chain polymer (larger than Si_5_O_16_) and that polymerization advanced with an increase of the synthesizing temperature [16]. Different polymorphs of calcium silicate hydrates with Ca/Si = 2.0, namely, *α*-C_2_SH [Ca_2_(SiO_4_H)OH], dellaite [Ca_6_Si_3_O_11_(OH)_2_], and hillebrandite, were synthesized and their stability and phase transformation were investigated under hydrothermal conditions up to 400°C. *α*-C_2_SH completely transforms to dellaite at 350°C for 1 hour which in turn undergoes a reversible phase transformation to hillebrandite at low temperatures below 300°C [17]. Hillebrandite was synthesized by mechanochemical treatment of amorphous precipitated silica/lime using a vibration mill at room temperature [18]. The second step involves calcination of hillebrandite to prepare belite. Hillebrandite starts to decompose at about 500°C producing low-crystalline *β*-C_2_S [15]. Moreover, ^29^Si MAS NMR analysis illustrates that the temperature at which *β*-C_2_S begins to form decreases as the Ca/Si ratio of hillebrandite becomes higher [19] or as the temperature of hydrothermal synthesis of hillebrandite becomes higher [16]. On the other hand, XRD analysis illustrates that the crystallographic properties and density of dissociation products of *α*-C_2_SH depend on the calcination temperature [20]. *α*-C_2_SH dissociates at 390–490°C forming an intermediate phase plus *γ*-C_2_S. The intermediate phase then transforms to *α*_L_′ phase at 920–960°C and yields *β*-C_2_S on cooling while hillebrandite yields *β*-C_2_S at the lowest temperature [21]. Hillebrandite synthesized by mechanochemical treatment of amorphous precipitated silica/lime decomposed into *β*-C_2_S below 1000°C [18].

The reactivity of *β*-C_2_S relates to its large specific surface area taking into consideration the fact that the hydration is chemical-reaction-rate-controlled until its completion [19]. The specific surface area of *β*-C_2_S depends on calcination temperature of hillebrandite. The hydration rate of *β*-C_2_S enhanced with increasing surface area. The specific surface area affects the hydration reaction mechanism. In the case of specific areas of 5.5 m^2^/g or less, the reaction changes from a chemical reaction to a diffusion-controlled one [22]. The hydration rate of *β*-C_2_S enhances with raising curing temperature of hydration [23]. The hydration rate of *β*-C_2_S enhanced with dynamic more than static hydration condition [24]. At early ages of hydration, *β*-C_2_S yields C–S–H with a Ca/Si ratio of 1.81 plus Ca(OH)_2_. At later ages, C–S–H and Ca(OH)_2_ react to form a C–S–H(II)-like monophase hydrate having a Ca/Si ratio of 1.98 with different morphology [24]. The hydration rate of the *β*-C_2_S also enhances with increasing water/solid ratio [25]. Recent works presented alternative cements containing higher amounts of *β*-C_2_S in their composition based upon the use of waste materials such as rice hull and husk ash as well as coal fly ash [15, 26, 27]. Silica fume is an abundant material produced in many countries as a waste product from silicon industry around the world containing approximately 90–97% os SiO_2_. Silica fume have average particle size <1 *μ*m, specific surface area of 13–30 m^2^/g, and specific gravity of 2.22. Reuse of waste materials seems to be a good approach to solve both economic and environmental issues related to cement production [28–30]. This work describes a method for the production of *β*-C_2_S from silica fume.

## 2. Materials and Experimental Techniques

Freshly prepared lime was prepared by calcination of limestone powder (purity > 99%) in an electrical muffle furnace at 950°C for 3 hours. Lime was cooled to room temperature in desiccator, milled, and stored in tightly closed plastic bottle to avoid carbonation. Silica fume was purchased from Ferrosilicon Company, Edfo, Egypt. Distilled water and analytical grade barium chloride were used without further purification. Preparation of hillebrandite by hydrothermal treatment of silica fume and lime with and without 2 wt% BaCl_2_ was carried out as described below. Mixtures of silica fume and lime with and without BaCl_2_ keeping the ratio Ca/Si or (Ca + Ba)/Si = 2.0 were hydrothermally treated at 110 or 150°C for 2 or 5 hours as illustrated in Table 1.

Mixtures of silica fume, lime, and BaCl_2_ were added to distilled water at water/solid ratio of 5/1 by weight in stainless steel capsule keeping the occupied volume equal to 0.67 of the total volume capacity. The capsule was tightly closed to avoid sealing of water vapor. The capsule was shacked to obtain homogeneous suspension inside. The capsule was heated in electric oven for appropriate temperature and time. At the end of the hydrothermal treatment process, the capsule was removed from oven and cooled to room temperature. The product of hydrothermal treatment (hillebrandite) was filtered, washed with distilled water, and dried under vacuum. Preparation of *β*-C_2_S was carried out by calcination of hillebrandite in an electric muffle furnace at 600 or 700°C for 3 hours. At the end calcination process, the calcined product (*β*-C_2_S) was cooled to room temperature in desiccator, milled, and stored in tightly closed plastic bottles. X-ray fluorescence XRF and X-ray diffraction XRD analyses were carried out by Philips X-ray diffractometer PW 1370 Co. with Ni filtered CuK_*α*_ radiation (1.5406 Å). The Fourier transform infrared FTIR analysis was measured by spectrometer Perkin Elmer FTIR System Spectrum X in the range of 400–4000 cm^−1^ with spectral resolution of 1 cm^−1^. Scanning electron microscopy SEM was investigated by Jeol-Dsm 5400 LG apparatus. The thermogravimetric TGA and differential thermogravimetric analyses DTG were carried out with the aid of Shimadzu Corporation thermoanalyzer with DTG-60H detector with 10°C/min heating rate from room temperature up to 1000°C; under nitrogen atmosphere at 40 mL/min flow rate, the hold time at the appropriate temperature is zero.

## 3. Results and Discussion

### 3.1. Characterization of Starting Materials

**Figure 2:**
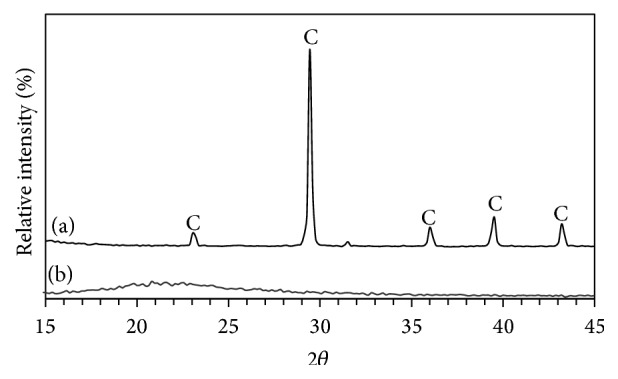
XRD patterns of (a) limestone and (b) silica fume (where C is calcite).

**Figure 3:**
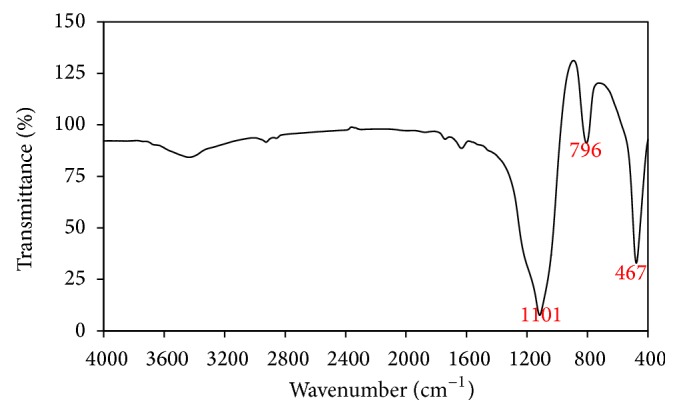
FTIR spectrum of silica fume.

**Figure 4:**
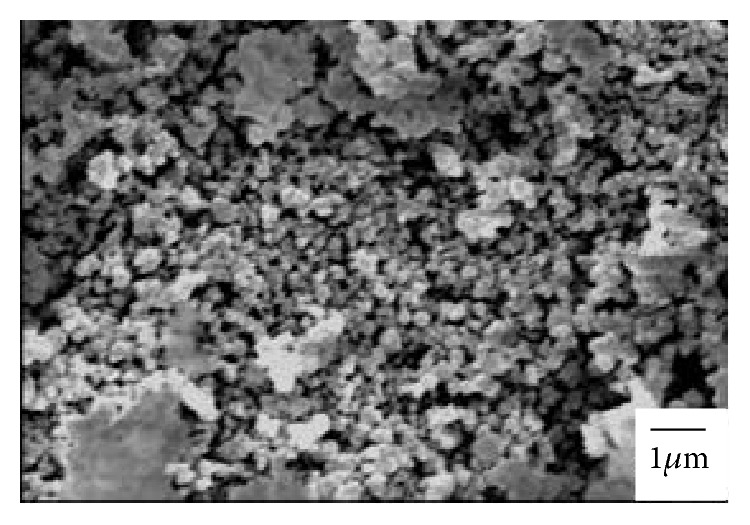
SEM micrograph of silica fume.

Figure 2 illustrates the XRD patterns of limestone and silica fume.  Table 2 illustrates the chemical composition of limestone and silica fume inferred by XRF. The chemical composition results confirm XRD results indicating that limestone is mainly composed of calcite (CaCO_3_) while silica fume is mainly composed of amorphous silica as indicated by a broad hump at around 20–25°2*θ*.  Figure 3 illustrates the FTIR spectrum of silica fume. The most significant absorption bands of silica appear at 1101, 796, and 467 cm^−1^ corresponding to asymmetric stretching vibration of Si–O–Si, symmetric stretching vibration of Si–O–Si, and bending vibration of O–Si–O, respectively [31].  Figure 4 illustrates the SEM micrograph of silica fume. Silica fume has a refined microstructure of high surface area containing silica microspheres.

### 3.2. Preparation of Hillebrandite

**Figure 5:**
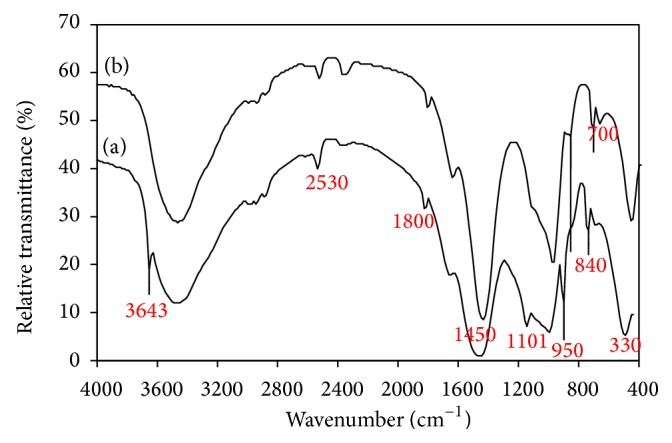
FTIR spectra of lime/silica mixture hydrothermally treated at 110°C for (a) 2 hours (b) 5 hours.

**Figure 6:**
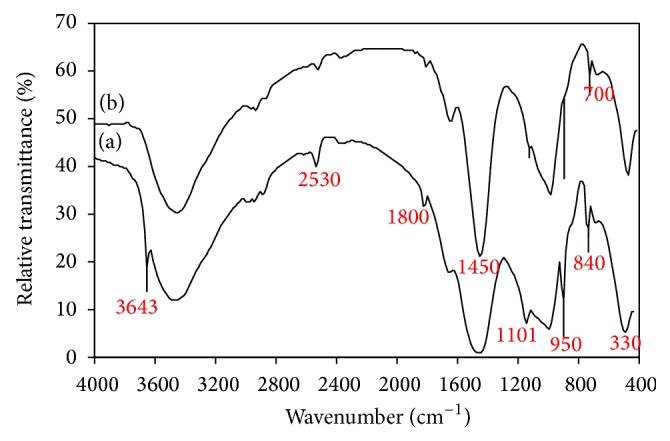
FTIR spectra of lime/silica mixture hydrothermally treated for 2 hours at (a) 110°C (b) 150°C.

Figures 5 and 6 illustrate the FTIR spectra of lime/silica mixture hydrothermally treated at 110–150°C for 2–5 hours. Hydrothermal treatment initiates the reaction between lime and reactive silica leading to the formation of intermediate calcium silicates hydrate (hillebrandite) which gives rise to strong absorption band at 950 cm^−1^ (vibrations of SiO_4_^−4^ groups) and strong absorption bands at 2530, 1800, and 330 cm^−1^ [32]. Unreacted silica gives rise to its absorption band at 1101 (Si–O–Si asymmetric stretching vibration) [31] and unreacted lime gives rise to its absorption band at 3643 and 1450–1500 cm^−1^ (vibrations of OH group and Ca–O stretching vibration) [32]. Calcite arising from partial carbonation of unreacted lime gives its absorption bands at 2530, 1800, 1420, 840, and 700 cm^−1^ [33]. The existence of unreacted lime and silica indicates that the hydrothermal treatment of the lime/silica mixture (Ca/Si = 2/1) at 110°C for 2 hours does not drive the reaction to completion. FTIR indicates that the chance of formation hillebrandite was improved by increase of both time (Figure 5) and temperature (Figure 6) of hydrothermal treatment. This is confirmed by disappearance of lime and the decreasing amount of unreacted silica. From economic point of view, the best conditions (heating at 110°C for 5 hours) were selected for the hydrothermal treatment of silica/lime mixtures.

**Figure 7:**
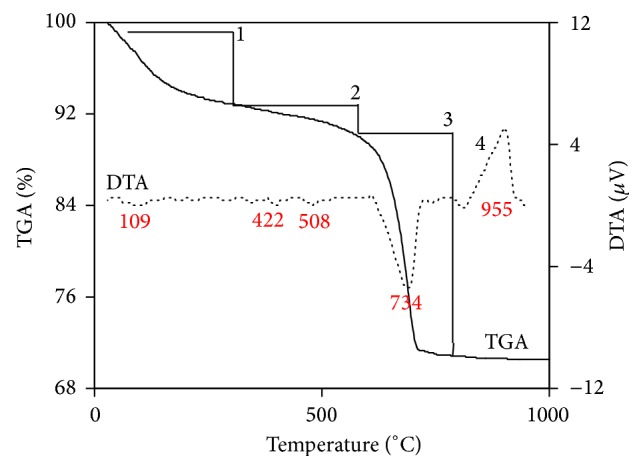
TGA/DTA thermogram of lime/silica mixture hydrothermally treated at 110°C for 5 hours.

Figure 7 illustrates TGA/DTA thermogram of lime/silica mixture hydrothermally treated at 110°C for 5 hours. Result of thermal analysis was illustrated in Table 3. Hillebrandite lost absorbed water at 109°C. Hillebrandite partially dehydrated in two steps at 422 and 508°C. Hillebrandite completely dehydrated forming *γ*-C_2_S at 734°C. Finally, *γ*-C_2_S transformed to *α*′-C_2_S at 955°C [34]. When allowed to cool to room temperature, *α*′-C_2_S may transform *β*-C_2_S instead of *γ*-C_2_S as illustrated in Figure 1. Fast cooling and presence of Ba^2+^ ions may stabilize *β*-C_2_S [13].

### 3.3. Preparation of Belite

**Figure 8:**
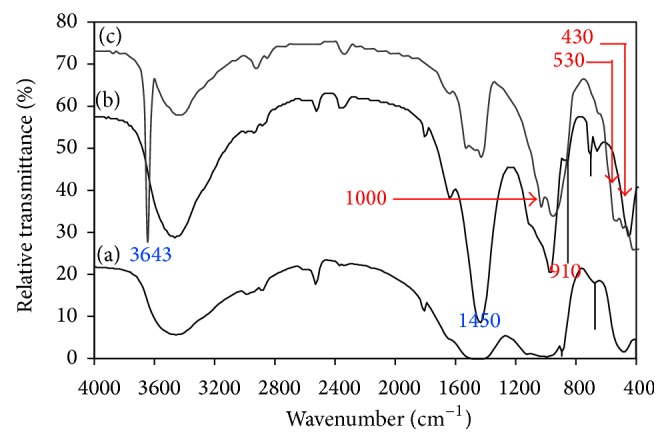
FTIR spectra of lime/silica mixture hydrothermally treated at 110°C for 5 hours (a) before calcination, (b) calcined at 600°C, and (c) calcined at 700°C.

Figure 8 illustrates the FTIR spectra of lime/silica mixture hydrothermally treated at 110°C for 5 hours and calcined at 600–700°C for 3 hours. As determined by TGA/DTA results, hillebrandite completely dehydrates forming *α*′-C_2_S that transforms to *β*-C_2_S and/or *γ*-C_2_S after cooling. C_2_S gives rise to the following absorption bands: three maxima at 1000, 910, and 840 cm^−1^ (Si–O asymmetric stretching modes), medium intensity band at 430 cm^−1^ (Si–O bending mode), and a strong band at 530 cm^−1^ (Si–O–Si out of plane bending mode) [35–37]. C_2_S does not clearly appear in sample that was calcined at 600°C (Figure 8(b)) but clearly appears in sample that was calcined at 700°C (Figure 8(c)). In general, formation of C_2_S was accompanied by liberation of lime (absorption bands at 3643 and 1450–1500 cm^−1^). This confirms the TGA/DTA results which show that hillebrandite completely dehydrated forming *γ*-C_2_S at 734°C.

**Figure 9:**
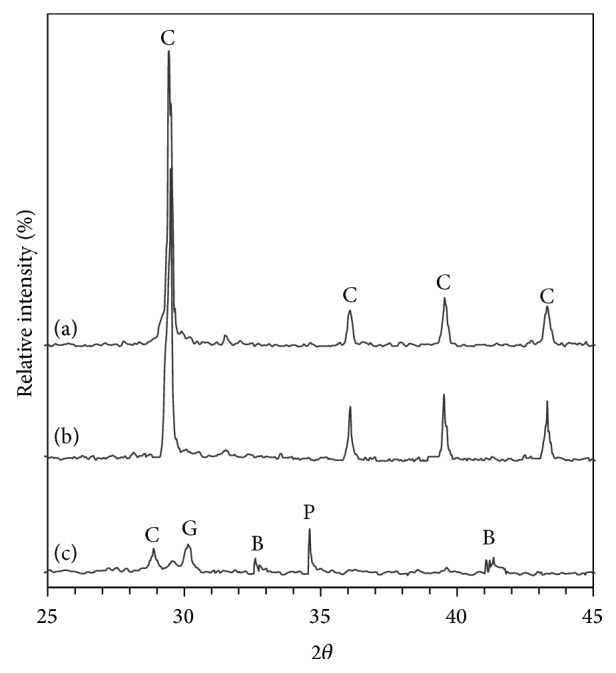
XRD patterns of lime/silica mixture hydrothermally treated at 110°C for 5 hours (a) before calcination, (b) calcined at 600°C, and (c) calcined at 700°C (where C is calcite, P is portlandite, G is *γ*-C_2_S, and B is *β*-C_2_S).

**Figure 10:**
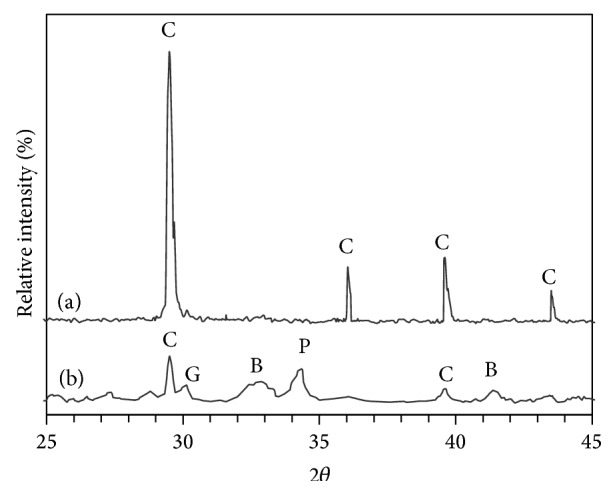
XRD patterns of (lime + BaCl_2_)/silica mixture hydrothermally treated at 110°C for 5 hours and then calcined for 3 hours at (a) 600°C and (b) 700°C (where C is calcite, P is portlandite, G is *γ*-C_2_S, and B is *β*-C_2_S).

Figure 9 illustrates XRD patterns of lime/silica mixture hydrothermally treated at 110°C for 5 hours calcined at 600–700°C for 3 hours. Hillebrandite does not appear in the XRD analysis (Figure 9(a)) because of its gel structure. C_2_S does not appear in the sample that was calcined at 600°C (Figure 9(b)). The mixture of *γ*-C_2_S (major) and *β*-C_2_S (minor) appears in the sample that was calcined at 700°C (Figure 9(c)). This proves that *β*-C_2_S was not stabilized by fast cooling and transforms to *γ*-C_2_S [11, 12].  Figure 10 illustrates XRD patterns of (lime + BaCl_2_)/silica mixture hydrothermally treated at 110°C for 5 hours calcined at 600–700°C for 3 hours. In this case, the situation is reversed. The mixture of *β*-C_2_S (major) and *γ*-C_2_S (minor) appears in the sample that was calcined at 700°C (Figure 10(b)). This indicates that *β*-C_2_S was stabilized by Ba^2+^ ions with minor transformation to *γ*-C_2_S. In other words, Ba^2+^ ions replace calcium and/or silicon atoms and hence it stabilizes the structure of belite and prevents the transformation of *β*-C_2_S to *γ*-C_2_S [13].

**Figure 11:**
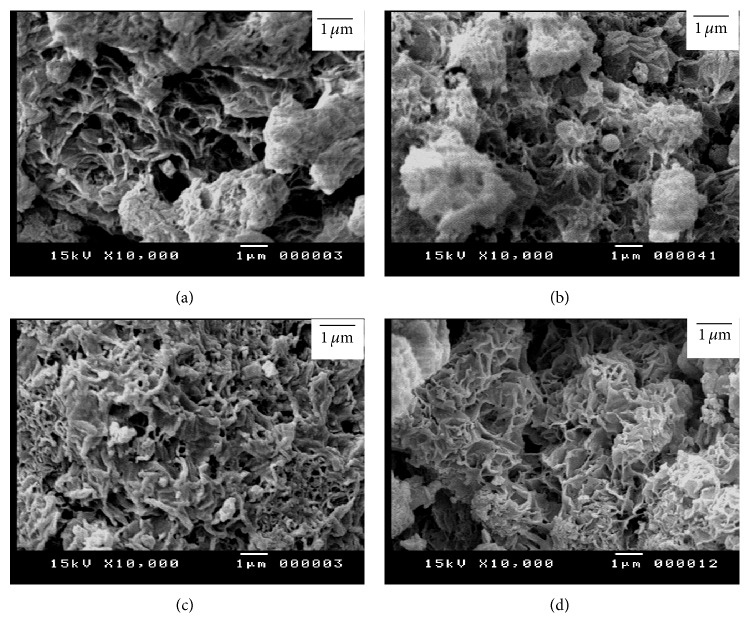
SEM micrographs of mixture of silica fume and lime hydrothermally treated at 110°C for (a) 2 hours without BaCl_2_, (b) 2 hours with BaCl_2_, (c) 5 hours without BaCl_2_, and (d) 5 hours with BaCl_2_.

**Figure 12:**
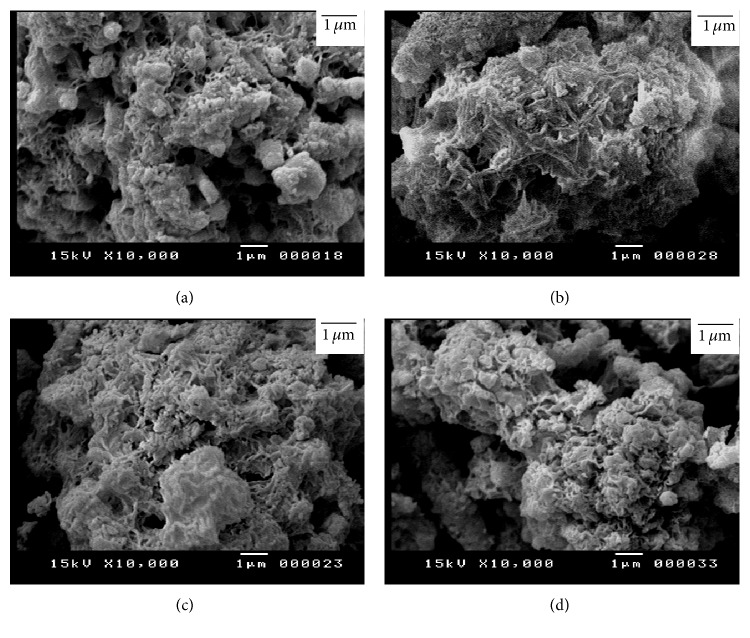
SEM micrographs of mixture of silica fume and lime hydrothermally treated at 150°C for (a) 2 hours without BaCl_2_, (b) 2 hours with BaCl_2_, (c) 5 hours without BaCl_2_, and (d) 5 hours with BaCl_2_.

**Figure 13:**
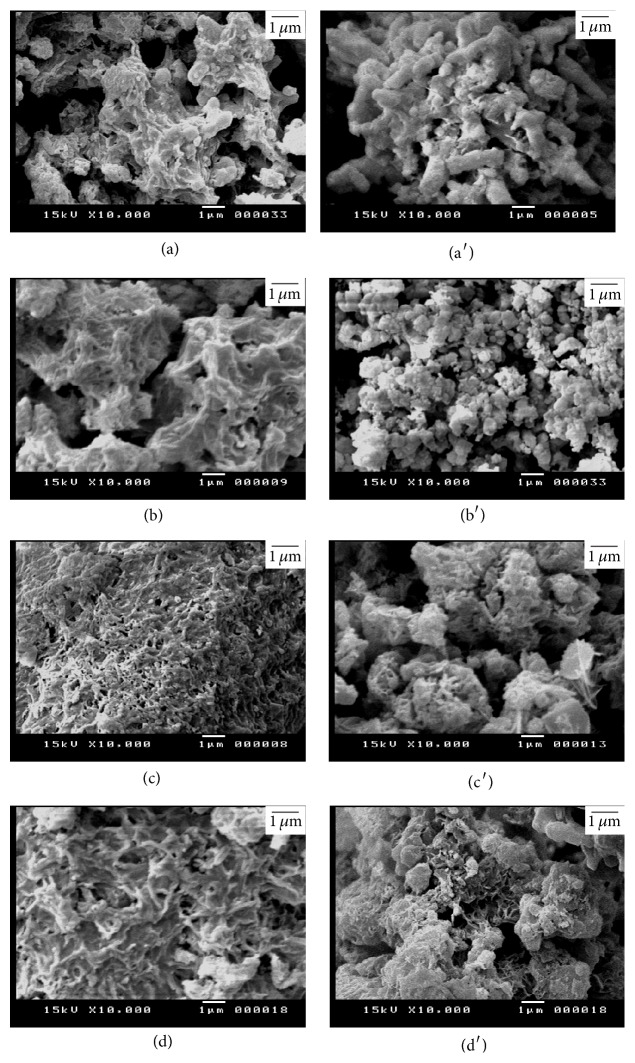
SEM micrographs of mixture of silica fume and lime represented in Figure 11 after being calcined at 600°C (right) and 700°C (left).

**Figure 14:**
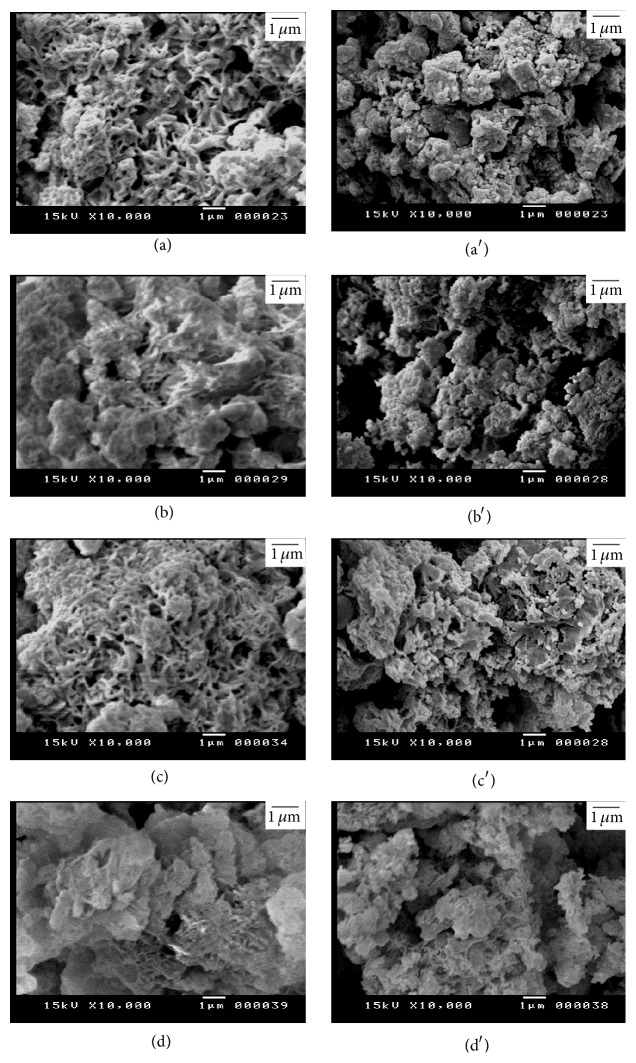
SEM micrographs of mixture of silica fume and lime represented in Figure 12 after being calcined at 600°C (right) and 700°C (left).

Figures 11 and 12 illustrate the SEM micrographs of mixture of silica fume and lime hydrothermally treated at 110–150°C for 2–5 hours. The mixtures of silica fume and lime that were hydrothermally treated at 110°C for 2 hours contain little amount of hillebrandite of low density with web-like morphology (Figures 11(a) and 11(b)). On the other hand, the mixtures that were hydrothermally treated at 110°C for 5 hours contain higher amount of hillebrandite of low density with web-like morphology (Figures 11(c) and 11(d)). The morphology of the hillebrandite greatly changes with raising the temperature of the hydrothermal treatment. The mixtures of silica fume and lime that were hydrothermally treated at 150°C for 2–5 hours with/without BaCl_2_ addition contain high amount of short fibrous hillebrandite of high density (Figures 12(a)–12(d)).  Figure 13 illustrates the SEM micrographs of mixtures of silica fume and lime hydrothermally treated at 110°C for 2–5 hours after being calcined at 600–700°C. As determined by TGA/DTA results, mixtures that were calcined at 600°C contain partially dehydrated calcium silicate with glass-like morphology (Figures 13(a)–13(d)). On the other hand, mixtures of silica fume and lime that were calcined at 700°C contain fine *β*-C_2_S crystals (Figures 13(a′) and 13(c′)). The amount of fine *β*-C_2_S crystals markedly increases in case of mixture of silica fume and lime with BaCl_2_ addition (Figures 13(b′) and 13(d′)). This confirms TGA/DTA results.  Figure 14 illustrates the SEM micrographs of mixtures of silica fume and lime hydrothermally treated at 150°C for 2–5 hours after being calcined at 600–700°C. Mixtures that were calcined at 600°C still contain partially dehydrated calcium silicate with glass-like morphology (Figures 14(a)–14(d)). The amount of fine *β*-C_2_S crystals formed in mixtures that were calcined at 700°C is not affected by raising the temperature of the hydrothermal treatment even in presence of BaCl_2_ addition (Figures 14(a′) and 14(d′)).

## 4. Conclusions

The following is concluded.

(1) FTIR results show that hillebrandite was successfully prepared by hydrothermal treatment of lime/silica fume mixtures with (Ca + Ba)/Si = 2 at 110°C for 5 hours.
(2) TGA/DTA results show that hillebrandite partially dehydrates in two steps at 422 and 508°C and transforms to *γ*-C_2_S at 734°C that in turn transforms to *α*′-C_2_S at 955°C.
(3) XRD results show that *α*′-C_2_S transforms to *β*-C_2_S upon cooling. In absence of Ba^2+^ ions, *β*-C_2_S undergoes major transformation to *γ*-C_2_S. Whereas, in presence of Ba^2+^ ions, *β*-C_2_S could be stabilized with formation of little amount of *γ*-C_2_S.
(4) Mixture of silica fume, lime, and BaCl_2_ with the ratio of (Ca + Ba)/Si = 2 was successfully utilized for synthesis of *β*-C_2_S by hydrothermal treatment at 110°C for 5 hours followed by calcination of the product at 700°C for 3 hours.

## Figures and Tables

**Table 1:** Mixtures of silica fume and lime with and without BaCl_2_ hydrothermally treated and calcined at different conditions.

Number	Hydrothermal treatment conditions
1	Silica fume, lime treated at 110°C for 2 hours
2	Silica fume, lime with BaCl_2 _treated at 110°C for 2 hours
3	Silica fume, lime treated at 110°C for 5 hours
4	Silica fume, lime with BaCl_2_ treated at 110°C for 5 hours
5	Silica fume, lime treated at 150°C for 2 hours
6	Silica fume, lime with BaCl_2_ treated at 150°C for 2 hours
7	Silica fume, lime treated at 150°C for 5 hours
8	Silica fume, lime with BaCl_2_ treated at 150°C for 5 hours

**Table 2:** The chemical composition of limestone and silica fume inferred by XRF.

Material	SiO_2_	Al_2_O_3_	Fe_2_O_3_	CaO	MgO	SO_3_	Na_2_O	K_2_O	L.O.I	Total
Limestone	0.26	0.16	0.00	54.59	0.29	0.05	0.11	0.03	43.72	99.21
Silica fume	92.90	1.10	0.82	0.42	0.52	0.00	0.64	1.12	1.56	99.08

**Table 3:** Result of thermal analysis.

Number	Phases	Temperature (°C)	Peak temperature (°C)	Weight loss (%)	Weight loss (mg)
1	Loss of absorbed water	30–287	109	6.85	0.555
2	Hillebrandite partial dehydration	288–590	422, 508	2.53	0.205
3	Hillebrandite complete dehydration (formation of γ-C_2_S)	593–781	734	19.6	1.60
4	γ-C_2_S → α′-C_2_S transformation	850–985	955	—	—

Total				28.98	2.36
